# Observation of water droplets in microporous layers for polymer electrolyte fuel cells by X-ray computed nano-tomography

**DOI:** 10.1107/S1600577522007949

**Published:** 2022-08-17

**Authors:** Satoshi Yamaguchi, Satoru Kato, Wataru Yoshimune, Daigo Setoyama, Akihiko Kato, Yasutaka Nagai, Takahisa Suzuki, Akihisa Takeuchi, Kentaro Uesugi

**Affiliations:** a Toyota Central R&D Labs, Inc., 41-1 Yokomichi, Nagakute-shi, Aichi 480-1192, Japan; b Japan Synchrotron Radiation Research Institute (JASRI)/SPring-8, 1-1-1 Koto, Sayo, Hyogo 679-5198, Japan; Tohoku University, Japan

**Keywords:** X-ray computed nano-tomography, polymer electrolyte fuel cell, gas diffusion layer, microporous layer, water droplet, hydro­phobic material

## Abstract

An X-ray computed nano-tomography (nano-CT) system has been established at the BL33XU beamline of SPring-8. The optical system consists of pseudo-Köhler illumination with a sector condenser zone plate, an apodization Fresnel zone plate as the objective lens, and a Zernike phase plate. The imaging detector is a fiber-coupling type X-ray camera. The performance of the X-ray nano-CT system was confirmed by imaging an X-ray test chart. The system was subsequently applied to the observation of a microporous layer for polymer electrolyte fuel cells and a simulated microporous layer including liquid water. The nano-CT system, which can perform a computed tomography measurement in less than 4 min, allowed visualization of a spherical water droplet produced in the microporous layer. In the present study, the shape of water droplets in a nanoscale porous structure is investigated.

## Abstract 2

Condensed liquid water was observed in the pores of a model microporous layer for a polymer electrolyte fuel cell using an X-ray computed nano-tomography system constructed at the BL33XU beamline of SPring-8. The water droplets had a spherical shape, regardless of the structure of the pores in the hydro­phobic porous material.

## 1. Introduction

Polymer electrolyte fuel cells (PEFCs) convert the chemical energy of hydrogen directly into electricity with the only by-product being water. PEFCs are a promising technology for achieving carbon neutrality. To increase the power density of PEFCs is one of the strategies for reducing the system cost. The key to increasing the power density is to manage liquid water that inhibits oxygen transport and electrochemical reactions in the cell (Hayashi *et al.*, 2017 ▸). PEFCs have a sandwich structure in which an electrolyte membrane coated with catalyst layers is located between gas diffusion layers (GDLs) and flow channels for the supply of reactant gases. The GDL typically consists of a substrate layer and a microporous layer (MPL). The substrate layer and MPL are commonly made of carbon fibers with diameters of several micrometres, and carbon nanoparticles, respectively. The substrate and MPL are generally hydro­phobized with polytetra­fluoro­ethyl­ene (PTFE). The water generated in the catalyst layer passes through the GDL and drains into the flow channel. The vapor condenses in the GDL when the PEFC is operated at a high current density or at a low temperature (Hayashi *et al.*, 2017 ▸; Sabharwal *et al.*, 2021 ▸; Naganuma *et al.*, 2012 ▸). However, the accumulation of a large amount of liquid water in the GDL inhibits oxygen transport, which results in a decrease in the cell potential. This phenomenon is referred to as flooding. Visualization of liquid water in a PEFC would contribute to strategies to avoid this flooding because it would allow better management and acceleration of drainage.

Many studies have investigated the liquid water transport behavior in the GDL of PEFCs. X-ray computed tomography (CT) has been used as a non-destructive observation method to visualize the liquid water distribution in PEFCs. Three-dimensional (3D) visualization of the dynamic behavior of liquid water in a GDL substrate during operation was investigated using *operando* X-ray micro-CT (Eller & Büchi, 2014 ▸; Nagai *et al.*, 2019 ▸; Kulkarni *et al.*, 2020 ▸). However, there is no literature on observations of the shape of liquid water droplets within the nanosize pores of an MPL due to the limited spatial resolution of X-ray micro-CT.

MPLs are known to suppress flooding in PEFCs (Gostick *et al.*, 2009 ▸). The through-plane distribution of liquid water in an MPL has been investigated by *operando* synchrotron X-ray radiography (Lee *et al.*, 2015 ▸; Antonacci *et al.*, 2016 ▸). Lee *et al.* reported the effects of the MPL thickness on the nature of water management in a PEFC performance using X-ray radiography. Although the MPL was observed to reduce the water at the interface between the catalyst layer and the GDL, only 2D visualization of the water distribution was possible. 3D visualization of the water distribution in an MPL has been attempted using synchrotron X-ray micro-CT (Kato *et al.*, 2020 ▸; Yamaguchi *et al.*, 2021 ▸). Kato *et al.* visualized the wet domain in the MPL of a GDL using synchrotron X-ray micro-CT under a water vapor supply to clarify water accumulation in the MPL. They demonstrated vapor condensation at the MPL and migration of water from the MPL through the substrate. However, they did not provide information regarding the detailed morphology of liquid water within the nanosize pores of the MPL. Yamaguchi *et al.* performed time-resolved X-ray micro-CT observations to elucidate the unsteady flow in the MPL during water injection. The study revealed water behavior in the large pores of the MPL; however, this observation of liquid water was performed in an MPL with pores with sizes of tens to hundred of micrometres.

It is also important to understand the accumulation and transport of water in the nanoscale pores of MPLs to reveal the mechanism by which MPLs suppress flooding. Although technology to achieve 3D observation of liquid water inside nanoscale pores of an MPL has not yet been realized, simulation studies of liquid water transport in nanoscale pores have been performed (Zenyuk *et al.*, 2015 ▸; Niblett *et al.*, 2020 ▸). For example, Niblett *et al.* conducted dynamic two-phase flow simulations to investigate the effect of GDLs with MPLs on liquid water percolation. The simulation and X-ray micro-CT results for water injection showed good agreement for the substrate and defects in the MPL. However, X-ray micro-CT has limited spatial resolution, and the simulation results could not be compared with the experimental results for the MPL. Therefore, we established a synchrotron-based nano-CT system with a high spatial resolution of at least 200 nm to observe liquid water within the nanosize pores of MPLs.

## 2. Construction of the nano-CT system

An X-ray computed nano-CT system was constructed at the BL33XU beamline [Toyota Beamline (Nonaka *et al.*, 2012 ▸, 2016 ▸)] of SPring-8 to clarify the water behavior in MPLs. The system is based on a full-field X-ray microscope and is widely used for non-destructive 3D imaging with a spatial resolution of 100 nm or higher. Nano-CT, which is used to observe nanoscale internal structures, has been developed and widely used at synchrotron radiation facilities and equipment manufacturers (see, for example, Martínez-Criado *et al.*, 2016 ▸; Ge *et al.*, 2018 ▸; Vandrade *et al.*, 2016 ▸; Flenner *et al.*, 2020 ▸; Stampanoni *et al.*, 2010 ▸; Carl Zeiss Microscopy, 2018 ▸).

**Figure 1:**
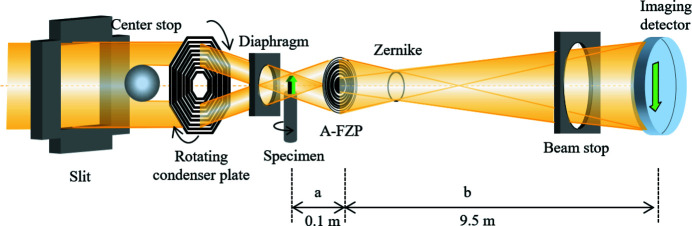
Schematic diagram of the Zernike phase-contrast X-ray microscopy optical system with pseudo-Köhler illumination constructed at SPring-8 BL33XU.

Fig. 1 ▸ shows a schematic illustration of the experimental setup. The system followed the nano-CT system of the BL47XU beamline at SPring-8. We employed pseudo-Köhler illumination by rotating a sector condenser zone plate [CZP; NTT Advanced Technology Corp. (NTT-AT), Japan] for sample illumination. The CZP had an octagonal shape with a 400 nm grating pitch, with 1.65 µm-thick tantalum as the zone material (Suzuki *et al.*, 2011 ▸). The CZP was set in a He-purged vessel mounted on a rotation stage. This illumination system was able to provide a uniform flat image field without speckle noise by rotating the CZP during exposure. A 300 µm-diameter diaphragm was used to select the first-order diffraction from the CZP. An apodization Fresnel zone plate (A-FZP; NTT-AT, Japan) made of tantalum was adopted as an objective lens, which realized Gaussian beam optics in the hard X-ray region (Takeuchi *et al.*, 2017 ▸). The zone thickness at the central region of the A-FZP is 1 µm and the zone depth in the outer region decreased gradually. The diameter of the A-FZP was 310 µm, the number of zones was 1550, and the outermost zone width was 50 nm. The focal length for 8 keV X-rays was 100 mm. Tantalum ring patterns, 0.96 µm thick with a diameter of 77.5 µm and a line-width of 4 µm, were used as the Zernike phase plate (ZPP; NTT-AT, Japan) designed for 8 keV X-rays of pseudo-Köhler illumination (Takeuchi *et al.*, 2009 ▸). The ZPP has pair of positive and negative ring patterns; a choice can be made between the bright phase-contrast mode and dark phase-contrast mode by selecting these ring patterns. The ZPP was placed at the back focal plane of the objective A-FZP. The imaging detector was a fiber-coupling type X-ray camera (C12849-101U, Hamamatsu Photonics, Japan) with high efficiency in the hard X-ray region (Uesugi *et al.*, 2017 ▸). The pixel size and the number of pixels for this imaging detector were 6.5 µm and 2048 × 2048, respectively. The maximum distance between the objective and the imaging detector was approximately 9.5 m. Helium gas was purged through the path of the X-ray beam to reduce the degradation and instability of the X-ray intensity. The wobble of the rotation stage (CRA070-002, Kohzu Precision, Japan) for the nano-CT measurement was less than 200 nm.

## 3. Experimental

Three performance tests of the X-ray nano-CT system were conducted at the Toyota beamline (BL33XU) of SPring-8. An absorption-contrast image and phase-contrast image of an X-ray test chart (XRESO-50HC, NTT-AT, Japan) were first obtained to evaluate the performance of the X-ray optical system. The energy of the incident X-rays was 8 keV, the X-rays were monochromated by a liquid-nitro­gen-cooled Si(111) double-crystal monochromator, and high-order harmonic X-rays were reduced using a double total-reflection Pt-coated mirror (5 mrad). For the illumination system of the X-ray microscope, pseudo-Köhler illumination with a rotating CZP was adopted. The magnification of the X-ray optics was ∼95 at 8 keV. The exposure time for the X-ray projection image was 100 ms.

X-ray nano-CT measurements were then performed on a cut piece of an MPL to evaluate the X-ray nano-CT system performance. The MPL specimen was stripped from a commercially available GDL (AvCarb GDS3260). This GDL was composed of a carbon-paper-based substrate and an MPL. The MPL specimen size required to obtain clear CT images was ∼70 µm × 70 µm × 70 µm, which is equivalent to the field of view of the X-ray nano-CT projection image width. The condition of the X-ray optics was phase-contrast mode with the ZPP used for the first performance test. A pulse-controlled rotation stage was used to rotate the sample by exactly 180°. The scan was performed using an on-the-fly method with 1800 projections over 180° of rotation. The scan time for 0.1° was 130 ms; the exposure time and data transfer time between X-ray camera and control PC were 100 ms and 30 ms, respectively, for each projection. The total time required for a CT measurement was 234 s. The CT reconstruction was conducted using a convolution back-projection method (Uesugi *et al.*, 2010 ▸). *Fiji* (Schindelin *et al.*, 2012 ▸) and *Geodict* (Math2Market GmbH) were used for volume rendering of the CT images.

**Figure 2:**
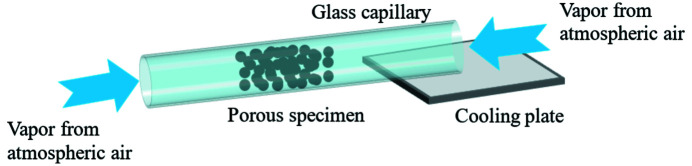
Schematic diagram of the condensation process of a simulated MPL. The length of the glass capillary was about 10 mm, and both ends of the glass capillary were open.

X-ray nano-CT measurements for visualization of liquid water in the MPL pores were then performed. The MPL specimen was a simulated MPL fabricated in a glass capillary (Yoshimune *et al.*, 2021 ▸). Nanosize carbon black particles (Vulcan XC-72R, Cabot Corp.) and PTFE particles (KTL-500F, Kitamura, Japan) in powder form were used. The primary particle diameters of the carbon black and PTFE were 50 nm and 500 nm, respectively. The carbon black and PTFE powders were mixed in a weight ratio of 60:40. The composite powder was filled into a glass capillary with an inner diameter of 60 µm. The capillary was annealed at 360°C for 30 min in air to melt the PTFE powder and coat the PTFE onto the carbon black particle surfaces. The specimen obtained by this process was a hydro­phobic porous carbon material that simulated an MPL. To supply liquid water into pores of the specimen, water vapor condensing in the air due to cooling was used, shown in Fig. 2 ▸. One end of the glass capillary of the specimen was in contact with a Peltier module to cool the entire specimen. The surface temperature of the Peltier module was set to −5°C when the Peltier module was operated. The water vapor in the glass capillary condensed on the specimen pores. The specimen was held on the sample stage of the X-ray nano-CT system after this condensation process. The conditions for the X-ray nano-CT measurement and the reconstruction process were the same as those for the second performance test.

## 4. Results and discussion

**Figure 3:**
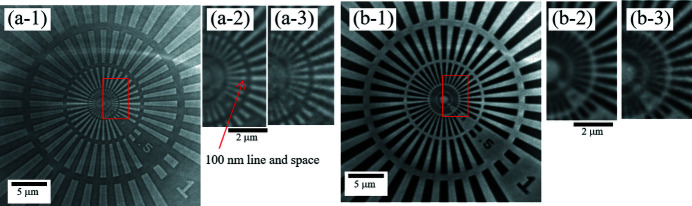
X-ray test chart (NTT-AT XRESO-50HC) images. (*a*) Absorption-contrast image, and (*b*) phase-contrast image with ZPP. Panels (*a*-1) and (*b*-1) show averaged 50-shot images of each with 100 ms exposure; (*a*-2) and (*b*-2) show enlarged images of the area indicated by the red squares in (*a*-1) and (*b*-1), respectively; (*a*-3) and (*b*-3) show single-shot images of the same positions of (*a*-2) and (*b*-2) with 100 ms exposure. The area indicated by the arrow corresponds to a 100 nm line and space, which can be clearly identified in both absorption- and phase-contrast images. Comparing the 100 ms single-shot and the 100 ms averaged 50-shot case, similar images were obtained with only a difference in the signal-to-noise ratio, which indicates good temporal stability.

Fig. 3 ▸ shows the first performance test results for X-ray images of the test chart. These are (*a*) absorption-contrast and (*b*) phase-contrast images of the average of 50 shot images with a 100 ms exposure. The effective pixel size was 67.2 nm with this optical system. Line-and-space patterns of 200 nm (100 nm line-width) were successfully observed in both contrast images. The spatial resolution of the X-ray optic system is thus higher than 200 nm. The 50 shot-averaged images of each contrast in Figs. 3 ▸(*a*-2) and 3(*b*-2) resemble the single-shot images in Figs. 3 ▸(*a*-3) and 3(*b*-3) in that the 200 nm lines and spaces can be identified. This indicates that the X-ray nano-CT optical system has temporal stability in the region below 0.5 Hz on the scale of 200 nm.

**Figure 4:**
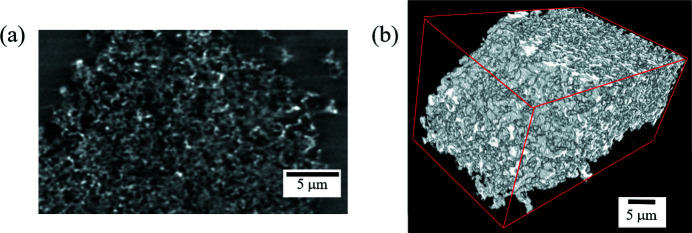
Nano-CT images of a cut MPL piece in dark phase-contrast mode. (*a*) Reconstructed CT image, and (*b*) 3D rendered image.

From the second performance test, Fig. 4 ▸ shows a CT image and a volume rendering of a CT image of a cut piece of the MPL. The projection images were taken in Zernike dark phase-contrast mode with the ZPP. In absorption-contrast mode, CT images were obtained in which the material and voids were distinguishable but had low contrast due to the small size of the specimen and the low X-ray absorption. In the CT image shown in Fig. 4 ▸(*a*), the dark gray domain represents air, while the white and light gray domains represent the solid materials of the MPL. The particles that compose the MPL were successfully visualized. Fig. 4 ▸(*b*) shows a 3D CT image of the MPL with a fine complex porous structure evident.

**Figure 5:**
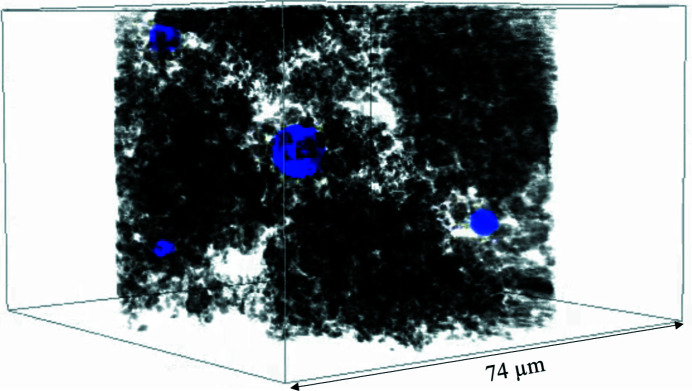
A 3D volume rendered image of the simulated MPL measured by X-ray nano-CT. Solid materials that comprise the hydro­phobic pore structure are represented in black. The blue color corresponds to liquid water. Four spherical-shaped liquid water areas were observed in the pores (see Movie S1 of the supporting information).

Fig. 5 ▸ shows the result of the third performance test, the observation of liquid water in the simulated MPL – a 3D volume rendered image of the entire field of view measured by phase-contrast mode X-ray nano-CT. The CT images were binarized and categorized into three categories: solid materials such as carbon black or PTFE, liquid water and voids. In the X-ray region, the phase-shift cross section is up to ∼1000 times larger than that of absorption for light elements (Momose & Fukuda, 1995 ▸). In phase-contrast mode, therefore, it is much easier to discriminate the solid materials, water and voids than in absorption mode. The solid material size ranged from a few hundred nanometres to a few micrometres, and some particles were agglomerated, while others were dispersed. The specimen had a porous structure with voids between the solid materials, and the porosity estimated from the X-ray nano-CT data was 81%. Since the spatial resolution of the nano-CT was 200 nm, the nano-CT measurement cannot observe pores smaller than 100 nm. The corrected porosity of the simulated MPL was 90%, assuming 10% volume fraction of pores less than 100 nm as in a general MPL (Matsuoka *et al.*, 2021 ▸). The liquid water was observed as spherical droplets that exist in the gaps between the porous materials. There are four water droplets within this field of view. The diameter of the water droplets ranged from 3.29 µm to 8.15 µm. The clear outline of the water droplet was thus successfully observed in the nanoscale hydro­phobic porous structure that simulates the MPL of PEFC.

**Figure 6:**
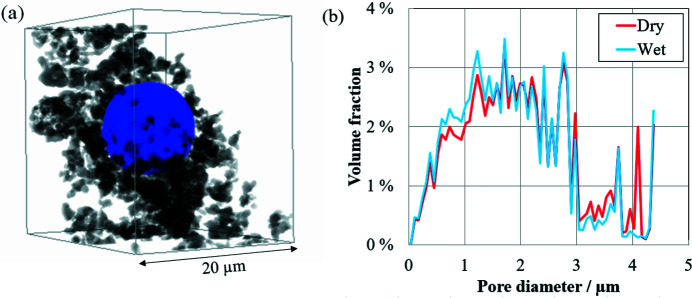
(*a*) 3D volume rendered image cropped around a 8.15 µm water droplet. (*b*) Pore size distribution obtained from the volume rendering image in (*a*) (see Movie S2 of the supporting information). The wet state indicates the distribution with a water droplet, while the dry state indicates that without a water droplet by removal after the binarization process.

Fig. 6 ▸(*a*) shows a 3D volume rendered image including the 8.15 µm water droplet shown in the central part of Fig. 5 ▸, cut out as a cube with dimensions of 20 µm per side. Fig. 6 ▸(*a*) visually shows the situation around the water droplet, which could not be confirmed in Fig. 5 ▸. Fig. 6 ▸(*b*) shows the pore size distribution in the region shown in Fig. 6 ▸(*a*). Two types of pore size distributions were obtained from Fig. 6 ▸(*a*) – the pore size distribution in the wet state with water droplets and that in the dry state without water droplets. The structure without liquid water can be taken to be the pore size distribution in the dry state because the images are segmented into three elements: solid material, water and voids. The major pore sizes in the pore size distribution were 1 µm to less than 3 µm. These pores correspond to the gaps where the particles of the material have agglomerated. While there were also pores larger than 3 µm, which correspond to gaps in the aggregated particle clusters, the main change in the pore size distribution in the wet state compared with that in the dry state was a reduction of the fraction of pores with a diameter of 4.1 µm. This is smaller than the droplet size of 8.15 µm. Next we explain this discordance.

**Figure 7:**
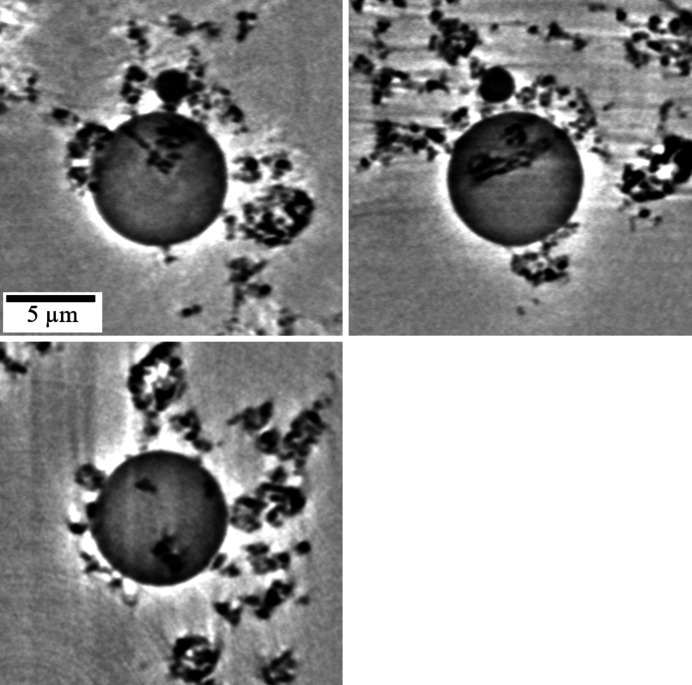
Orthogonal-cut images of the 3D volume rendered image in Fig. 6 ▸(*a*) without binarization. The water droplet appears as a circle in the center of each cross-sectional image. The water droplet includes hydro­phobic solid materials.

Fig. 7 ▸ shows orthogonal-cut images of Fig. 6 ▸(*a*) without binarization. The dark gray circular domains observed at the center of the *XY*, *XZ* and *YZ* planes represent water droplets. Solid mater­ials appear as black areas. In the case of a hydro­phobic porous structure, liquid water tends to be present in large pores (Cummins *et al.*, 2017 ▸). The hydro­phobic material has a large contact angle (>90°); therefore, it is assumed that liquid water follows the structure of the large pores. However, the present observation shows that liquid water is in a spherical form, regardless of the pore morphology. All four droplets, which are observed in Fig. 5 ▸, were found to include solid hydro­phobic materials [Fig. 6 ▸(*a*)]. Part of the material forming the pore structure in the dry state was hidden by water droplets, which resulted in a decline in the reduction of the fraction of pores with a diameter of 4.1 µm in the wet state. This discordance can be explained by the presence of particles submerged in the liquid water.

**Figure 8:**
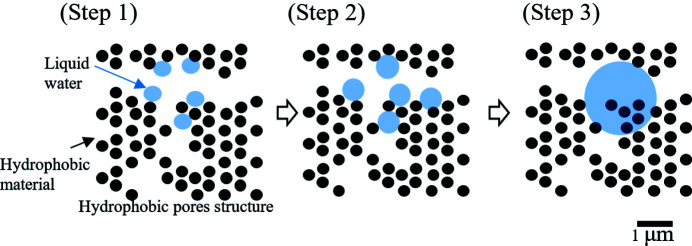
Schematic images of water droplet growth and aggregation model, which is the mechanism for the formation of liquid water in a hydro­phobic pore structure. (Step 1) Liquid water droplets less than 1 µm in diameter condense at locations in the hydro­phobic porous material. (Step 2) The water droplets grow with a spherical shape. If there are water droplets nearby, then they possibly coalesce when they come into contact with each other. (Step 3) The spherical shape of the liquid water droplets is stable, and the droplets surround hydro­phobic material.

The mechanism for the formation of liquid water in a hydro­phobic pore structure is discussed according to the observation results where the water droplets are spherical and contain solid materials. Fig. 8 ▸ shows a new model for the mechanism for the formation of water droplets. The steps are as follows.

Water droplet growth and aggregation:

(1) Spherical liquid water regions with diameters of less than 1 µm will condense at locations in the hydro­phobic porous material where condensation is likely to occur.

(2) The water droplets grow in spherical form. If there are water droplets nearby, then they possibly coalesce when they come into contact with each other.

(3) The spherical form of the liquid water is stable. The water droplet grows with the inclusion of the hydro­phobic mater­ial, while maintaining its spherical form.

This model supports the result that the liquid water droplets are spherical and include solid materials. We assume that this model is a match for water droplets with a limited scale on the submicrometre to 5 µm in a hydro­phobic pore structure. A water droplet larger than this is not necessarily expected to maintain a spherical shape in a hydro­phobic pore structure, because of the relationship between the surface tension of the water droplet and the hydro­phobic properties of the porous material. The possibility that the surface of the carbon particles was hydro­philic cannot be entirely ruled out, because the carbon was annealed in an air atmosphere. Water droplets may thus form around the hydro­philic surface of carbon particles. In this observation, the condensation of liquid water in an MPL was revealed as to be expected during the early stage of power generation. The results provide information that can facilitate the improvement of drainage from the MPL of a PEFC.

## 5. Summary

An X-ray computed nano-tomography system was constructed at the BL33XU beamline of SPring-8. The effective pixel size with this system was 67.2 nm at 8 keV. The spatial resolution of the optical system was higher than 200 nm and the measurement time for nano-CT was less than 4 min.

The particles that compose the MPL were successfully visualized by the nano-CT system in Zernike phase-contrast mode. 3D nano-CT imaging will provide information on the characteristics of porous structures (*e.g.* pore size and tortuosity) to conduct computer simulations of transport phenomena.

Condensed liquid water was observed in the pores of a model MPL. The water droplets had a spherical shape, regardless of the structure of the pores of the hydro­phobic porous material, and surrounded hydro­phobic material. The results presented here provide information that will contribute to the improvement of the drainage from the MPLs of PEFCs.

## Supplementary Material

Movie S1: 3D volume rendered image of the simulated MPL measured by X-ray nano-CT (Fig. 5 in the main manuscript). DOI: 10.1107/S1600577522007949/mo5256sup1.avi

Movie S2: 3D movie of image cropped around a 8.15 µm water droplet. [Fig. 6(a) in the main manuscript]. DOI: 10.1107/S1600577522007949/mo5256sup2.avi
